# Association of parathyroid hormone with risk of hypertension and type 2 diabetes: a dose-response meta-analysis

**DOI:** 10.1186/s12872-023-03682-1

**Published:** 2024-01-03

**Authors:** Mingming Feng, Mengqi Xu, Qing Wang, Shicui Xia, Cheng Yu, Min Li, Yafeng Liu

**Affiliations:** Nanjing Zijin Hospital, Nanjing, 211100 Jiangsu China

**Keywords:** Parathyroid hormone, Hypertension, Type 2 diabetes

## Abstract

**Background:**

Despite an increase in parathyroid hormone (PTH) has been reported to be associated with a higher risk of hypertension and type 2 diabetes (T2D), the comprehensive evaluation of the dose-response relationship between PTH and hypertension and T2D remains ambiguous. Therefore, a dose-response meta-analysis was performed to quantitatively investigate this association.

**Methods:**

PubMed, Web of Science, and Embase were systematically searched up to May 2023. Random-effect models were used to estimate the summary odds ratios (ORs) and 95% confidence intervals (CIs). Restricted cubic splines were used to model the dose-response association.

**Results:**

Ten articles (including 13 studies) were identified, with a total of 11,878 cases and 51,234 participants in the meta-analysis. Of these studies, eight (five cohort and three cross-sectional) studies investigated the association of PTH with hypertension; five (two cohort and three cross-sectional) studies assessed the association of PTH with T2D. The results showed a positive relationship between PTH and the risk of hypertension (OR,1.24, 95% CI: 1.16–1.33). We found a linear association between PTH and hypertension (*P*_non−linearity_= 0.222). In the dose-response analysis, the risk of hypertension increased 5% for every 10 pg/ml increase in PTH (OR,1.05, 95% CI: 1.02–1.08). The pooled OR of T2D risk for a 10 pg/ml increase in PTH was 1.00 (95% CI: 0.98–1.02).

**Conclusions:**

Elevated PTH is associated with an increased risk of hypertension. However, the evidence of the association between PTH and T2D is limited, and more well-designed studies need to be explored.

**Supplementary Information:**

The online version contains supplementary material available at 10.1186/s12872-023-03682-1.

## Background

The increasing incidence of hypertension and type 2 diabetes (T2D) has become an important public health problem in China. Hypertension is an important risk factor that in turn promotes the occurrence and development of T2D [1]. It is predicted that the number of people with hypertension and T2D will increase to 642 million and 1.56 billion respectively in 2025 and 2040 [2, 3]. The treatment of hypertension and T2D poses a great challenge to current medical care. Unfortunately, the specific pathogenesis of hypertension and T2D has not yet been fully elucidated. The development of hypertension and T2D are considered to be associated with many risk factors. Identification of these modifiable risk factors is an important step toward the prevention and management of hypertension and T2D.

Parathyroid hormone (PTH) mainly regulates cyclic calcium concentrations via enhancing bone resorption, inhibiting loss of urinary calcium, and promoting calcitriol formation (Vitamin D metabolites) [4]. Evidence exists that elevated PTH is associated with beta cell exhaustion, insulin resistance, and abnormal blood sugar [5–8]. Moreover, PTH activates the renin-angiotensin-aldosterone system, leading to an increase in renin release, which eventually leads to an increase in blood pressure [9]. Although the above association has been discovered, the closer ties have long been controversial. Thus, a comprehensive mastery of the effect of PTH on the above diseases is warranted.

Yao et al. examined the relationship between PTH and incident hypertension among 7504 Atherosclerosis Risk in community participants without initial hypertension and found that PTH may be associated with hypertension in blacks [10]. Similarly, Kim et al. suggested that serum PTH may be an independent risk factor for hypertension in middle-aged and older Korean adults [11]. Elevated PTH has also been found to be linked to an increased risk of diabetes [8, 12]. Nevertheless, no comprehensive assessment of the quantitative dose–response association between PTH and hypertension and T2D risk has been reported. Therefore, we performed a systematic review and meta-analysis of all available data from cross-sectional and cohort studies on the dose–response association of PTH and risk of hypertension and T2D.

## Methods

PubMed, Web of Science and Embase were searched up to May, 2023 for English articles examining the association between PTH and hypertension and T2D. The specific search strategies are shown in Supplementary Table 1. We also manually screened related bibliographies and review articles from other publications.

Studies were included if: (1) study participants were adults (≥ 18 years); (2) study types were cohort studies or cross-sectional studies; (3) the report provided relative risks (RRs), odds ratios (ORs), or hazard ratios (HRs) with 95% confidence intervals (CIs), or data to calculate these data; (4) the article provided PTH stratification, number of cases, exposed person-years, or number of participants.

Exclusion criteria were (1) reviews, letters, or case reports; (2) non-human studies; (3) insufficient details of PTH dose; (4) PTH was reported as a dichotomous variable; (5) duplicate data. If multiple articles based on the same study were published, we chose those with the most informative reporting of PTH levels and/or the larger sample size.

Two authors (M.F. and M.X.) extracted data on the first author, publication year, country, study design, characteristics of participants (sex and age), follow-up years, sample size, number of cases, total persons or person-years per PTH category, the definition of hypertension and T2D, quality assessment, ORs/RRs/HRs with 95% CIs, and covariates adjusted for analysis. The cohort study qualities were assessed by the Newcastle-Ottawa Scale. It includes eight aspects of the study, with an overall score of up to 9 [13]. We adopted the Agency for Healthcare Research and Quality (AHRQ) to evaluate the qualities of the cross-sectional study, which involved 11 aspects [14]. Any disagreements were resolved until we reached an agreement.

We calculated according to the total number of cases and the reported effect size in the absence of a number of cases in each layer of PTH. If the number of participants in each layer of PTH was missing, then the number of participants in each layer was assumed to be equal. We considered two separate studies if an article reported both hypertension and T2D. If the median or mean of PTH concentration for each category was unknown, then the midpoint of the upper and lower boundaries of each layer was regarded as the mean. If the highest category for PTH concentration was open ended, the width of the interval was assumed to be the same as in the closest category. When the lowest category was open ended, the lower boundary was set to 0 [15]. *P* values and heterogeneity were calculated by Cochrane Q method and *I*^*2*^ statistics, respectively. *P* < 0.05 was considered statistically significant; *I*^*2*^ values of about 25%, 50% and 75% represent low, medium and high heterogeneity, respectively; The random effect model was used for *I*^2^ statistics > 50%, and the fixed effect model was used for the contrary. We used generalized least square regression to estimate the dose-response relationship. Study-specific OR estimates were calculated per 10 pg/ml increment of PTH and then pooled. By modeling PTH with restricted cubic splines (three nodes at the 25th, 50th, and 75th percentiles of the distribution), the nonlinear correlation was tested. The nonlinear *P*-values were calculated by testing the null hypothesis that the quadratic spline coefficient was equal to zero.

Subgroup analysis stratified by country, follow-up time, gender, study design, outcome definition, detection method, and sample size. One study at a time was excluded for sensitivity analysis to assess the stability of the results and the source of heterogeneity. Egger’s test and Begg’s test evaluated whether there was publication bias.

## Results

**Fig. 1:**
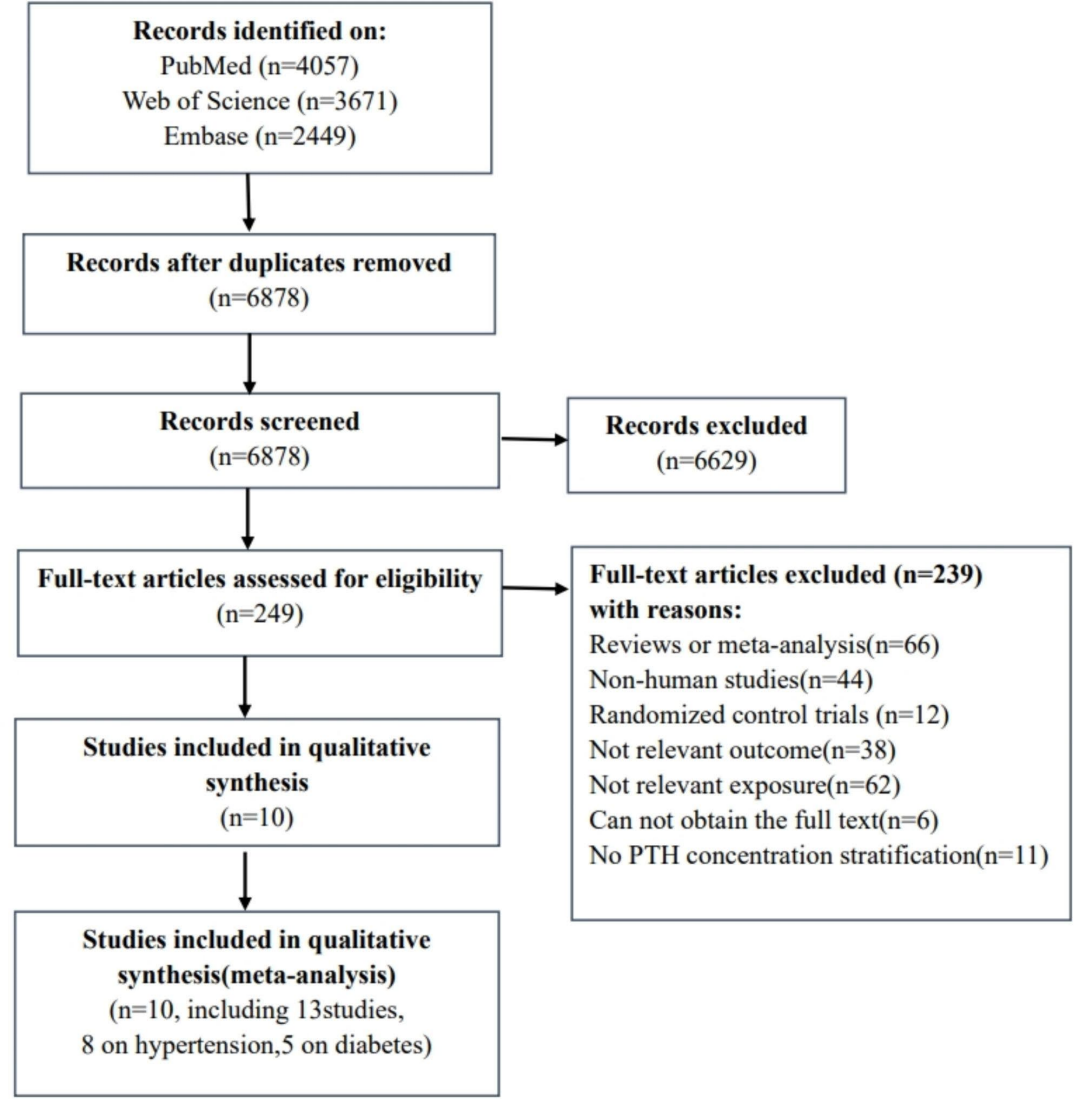
Flow chart of article selection

In PubMed, Web of Science and Embase, we identified a total of ten articles (13 independent studies), among which eight (five cohort, three cross-section) were about the studies on PTH and hypertension [10, 16–22], and five (two cohort, three cross-section) on PTH and T2D [8, 16, 18, 22, 23]. The specific screening process are shown in Fig. 1. The main characteristics of the cohort and cross-sectional studies are in Supplementary Tables 2 and Table 3. Overall, ten of the studies were conducted in the United States, two in South Korea and one in the Netherlands. Eight studies with 9135 cases and 28,433 individuals assessed the association between PTH and risk of hypertension. Five studies with 2743 cases/22,801 participants were identified in the dose-response analysis of PTH and T2D. Quality analysis of cohort studies and cross-sectional studies are shown in Supplementary Tables 4 and 5, respectively. Six articles defined hypertension as Systolic blood pressure (BP) ≥ 140 mmHg or diastolic BP ≥ 90 mmHg or the use of antihypertensive medications; One as self-reported hypertension; And the other article hypertension was determined by the definition of the international classification of diseases. For the definition of T2D, two articles were based on patients’ self-report, use of any T2D medication, or a blood glucose level ≥ 7 mmol/L, two articles were based on the criteria of hyperglycemia, and one article was based on the international disease classification.

When comparing high versus low PTH level, the pooled OR for hypertension was 1.24 (95% CI: 1.16–1.33, *I*^*2*^ = 39.6%, *P*_heterogeneity_=0.115; Supplementary Fig. 1). We found no evidence of publication bias by Egger’s test (*P* = 0.198).

**Fig. 2:**
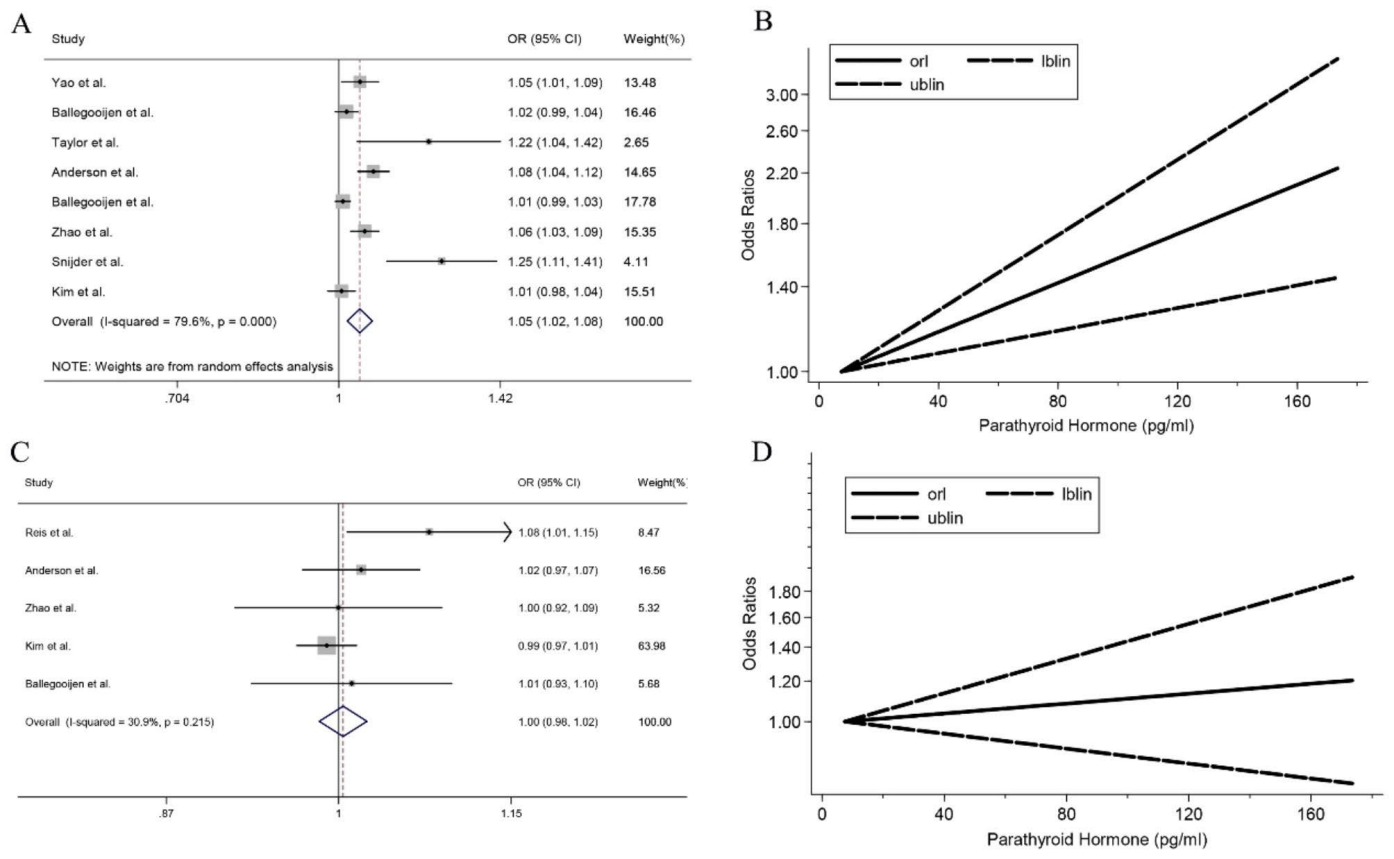
Dose-response association of PTH and hypertension and T2D. PTH, parathyroid hormone; T2D, type 2 diabetes. (A) Forest plot of study-specific odds ratio statistics for hypertension per 10 pg/ml increment of PTH; (B) Linear dose-response association between PTH and hypertension; (C) Forest plot of study-specific odds ratio statistics for T2D per 10 pg/ml increment of PTH; (D) Linear dose-response association between PTH and T2D

Data from five cohort and three cross-sectional studies were included in the linear dose-response analysis of PTH. With each 10 pg/ml increase in PTH concentration, the risk of hypertension was increased 5% (OR, 1.05, 95% CI: 1.02–1.08, *I*^2^ = 79.6%, *P*_heterogeneity_<0.001; Fig. 2A). We adopted restricted cubic splines to simulate the linear dose-response relationship. The results showed a positive linear correlation between PTH level and hypertension risk (*P*_nonlinearity_ =0.222, Fig. 2B). Sensitivity analysis results indicated that ORs ranged from 1.02 to 1.04, and our results were statistically stable (Supplementary Fig. 2A). Begg’s test (*P* = 0.048) and Egger’s test (*P* = 0.023) showed a significant publication bias (Supplementary Fig. 2B). The trim and fill method used to adjust for publication bias, the main result was not altered (OR: 1.03, 95% CI: 1.02–1.04).

The pooled OR of T2D for the highest versus lowest concentration of PTH was 1.10 (95% CI: 0.92–1.31, *I*^2^ = 0.0%, *P*_heterogeneity_=0.561; Supplementary Fig. 3). Publication bias was not detected (Egger’s test, *P* = 0.169).

Data from two cohort and three cross-sectional studies were included in the linear dose-response analysis of T2D. The pooled OR for T2D was 1.00 (95% CI: 0.98–1.02, *I*^2^ = 30.9%, *P*_heterogeneity_=0.215; Fig. 2C). Restricted cubic splines were used to simulate the linear dose-response relationship, there was no evidence of a nonlinear association between PTH and T2D (*P*_nonlinearity_ =0.417), we failed to obtain a significant dose-response relationship between PTH and T2D (Fig. 2D). The results were invariant in sensitivity analysis, and the stability ranged from 0.98 to 1.02 in the overall analysis (Supplementary Fig. 4A). Egger’s test (*P* = 0.237) showed no publication bias (Supplementary Fig. 4B).

Table 1 showed the subgroup analysis results of PTH and hypertension, which explored potential sources of heterogeneity. The summary OR for cohort studies was 1.05 (95% CI: 1.02–1.08; *P* = 0.003) and for cross-sectional studies was 1.07 (95% CI: 1.00-1.15; *P* = 0.055). Overall, the association was consistent across most analyses. The heterogeneity of electrochemiluminescence immunoassay was lower, with *I*^2^ decreased from 79.6 to 39.9%.

**Table 1:** Dose-response subgroup analysis of the risk of hypertension and type 2 diabetes with parathyroid hormone

Characteristics	Hypertension					type 2 diabetes			
	n	OR (95% CI)	I^2^ (%)	*P* ^*^		n	OR (95% CI)	I^2^ (%)	*P* ^*^
Country									
USA	6	1.07(1.04, 1.10)	65.4	0.013		4	1.03(1.00, 1.06)	0	0.465
Netherlands	1	1.01(0.99,1.03)	-	-		-	-	-	-
Korean	1	1.01(0.98, 1.04)	-	-		1	0.99(0.97, 1.01)	-	-
Detection method									
electrochemiluminescence immunoassay	3	1.06(1.02, 1.10)	39.9	0.189		2	1.05(0.99, 1.10)	44.9	0.178
chemiluminescence assay	4	1.03(1.00, 1.06)	81.2	0.001		3	1.00(0.98, 1.02)	0	0.558
immunoradiometric assay	1	1.05(1.02, 1.08)	-	-		-	-	-	-
Sample size									
> 3000	4	1.04(1.01, 1.06)	72.3	0.013		4	1.03(1.00, 1.06)	0	0.465
≤3000	4	1.10(1.02, 1.19)	86.3	0		1	0.99(0.97, 1.01)	-	-
Follow-up									
> 3	4	1.04(1.01, 1.07)	74.4	0.008		1	1.08(1.01, 1.15)	-	-
≤3	1	1.08(1.04, 1.12)	-	-		1	1.02(0.97, 1.07)	-	-
Sex									
M/W	7	1.05(1.02, 1.07)	80.2	0		-	-	-	-
M	1	1.22(1.04, 1.42)	-	-		-	-	-	-
Design									
cohort	5	1.05(1.02, 1.08)	79.1	0.001		2	1.04(1.00, 1.08)	42.8	0.186
cross-sectional	3	1.07(1.00, 1.15)	86.8	0.001		3	0.99(0.97, 1.01)	0	0.882
Hypertension definition									
measured	7	1.05(1.02, 1.07)	80.2	0		-	-	-	-
self-reported	1	1.22(1.04, 1.42)	-	-		-	-	-	-
Definition									
diabetes	-	-	-	-		3	1.03(1.00, 1.07)	3.7	0.354
hyperglycaemia	-	-	-	-		2	0.99(0.97, 1.01)	0	0.831

OR: odds ratio; CI: confidence interval; M: men; W: women

^*^P for heterogeneity within each subgroup estimated by the Cochran Q test

As for the subgroup analysis results of PTH and T2D, the summary OR for cohort studies was 1.04 (95% CI: 1.00–1.08; *P* = 0.133) and for cross-sectional studies was 0.99 (95% CI: 0.97–1.01; *P* = 0.431). The results were mostly consistent, except for the follow-up time study(≥ 3years) (OR, 1.08, 95% CI: 1.01–1.15, *P* = 0.031).

## Discussion

Our study provided the first systematic review and meta-analysis of cohort and cross-sectional studies of the dose-response relationship between PTH and hypertension and T2D. This meta-analysis, involving 51,234 participants, showed a linear and positive correlation between PTH and hypertension, each 10 pg/ml increase in PTH concentration was associated with a 5% increase in the risk of hypertension (OR, 1.05, 95% CI: 1.02–1.08). No evidence of the relationship between PTH and T2D was found.

The results acquired were aligned with a former meta-analysis, which also found a positive association between PTH concentration and the risk of hypertension [24]. However, different from the traditional binary analysis, we explored the dose-relationship quantitatively. Moreover, the present meta-analysis involved more comprehensive studies and larger sample size, which improved the reliability of the results. Meanwhile, several cohort and cross-sectional studies also found a positive association between PTH and the risk of hypertension, the results from our linear model examining hypertension risk suggest likewise [5, 11, 17, 18, 25, 26]. Notably, a cohort study found that elevated PTH levels, overall, were not independently associated with the risk of hypertension, but the relationship was observed among blacks not white [10]. The inconsistency might be explained by the racial differences and the small sample size, so further studies should consider potential interactions between races and explore ethnic differences in etiology. We found no evidence of the association between PTH and T2D in this meta-analysis, in contrast, in a prospective cohort study [8], elevated PTH concentration was independently associated with risk of T2D among whites. The discrepancy might be explained by that our meta-analysis included only 5 studies, of which three were cross-sectional studies.

In order to explore the high heterogeneity in this meta-analysis, we conducted subgroup analyses to identify the potential sources of heterogeneity. Results were consistent across most analyses. The results indicated that there was no statistical significance between PTH and hypertension in chemiluminescence assay and immunoradiometric assay, but statistical significance was observed in the electrochemiluminescence immunoassay. It hinted that the detection method of PTH may impact the relationship between PTH and hypertension. Moreover, we found that the association between PTH and risk of hypertension was not statistically significant in Korean and Netherlands. The physiology of markers of mineral metabolism, such as PTH and calcium, is intricately intertwined and may vary by race [27, 28], so within-subject biological variations are very significant, and thus impedes clinical application of PTH as a predictor of hypertension. To sum up, racial differences in these interrelationships may underlie the different findings in our study.

How parathyroid hormone causes hypertension may be related to the following mechanisms. First, PTH can stimulate the release of renin from glomeruli and activate the renin-angiotensin system, leading to increased blood pressure [29]. Second, elevated serum PTH levels activate adenylate cyclase on the cell membrane, generating cyclic adenosine monophosphate, which causes the intracellular mitochondrial pool to release Ca2 + into the cytoplasm, elevating intracellular free Ca2+, causing vascular smooth muscle contraction, which in turn constricts peripheral vasculature, causing elevated blood pressure [30]. Third, PTH may promote vascular calcification [31] and decrease the compliance of vessels, causing the important cushioning function of these arteries to be lost, which is known to result in increased pulse pressure and eventually induces hypertension [32].

Our finding may have great significance to public health. The prevalence of T2D and hypertension worldwide is rapidly increasing, and the role of PTH has not yet attracted public attention. This meta-analysis suggests that higher PTH concentrations might play a role in the pathogenesis of developing hypertension, these associations may underlie the increased risk for cardiovascular disease among participants with high PTH. Moreover, the level of PTH is closely related to serum calcium concentrations, and multiple epidemiological studies have shown that low levels of dairy calcium intake may be related to the high prevalence of hypertension [33–36]. Therefore, there is a reasonable biological link between PTH and the increase in blood pressure. It is worth noting that the average dietary calcium intake in most Asian countries is less than 500 mg/day [37], which falls far short of the recommended intake. It reminds us that increasing the intake of calcium in our daily life has a clear positive significance in preventing hypertension.

Our meta-analysis has some advantages. First, this is the first meta-analysis to evaluate the dose-response relationship between PTH and hypertension and T2D. Second, this study has a larger sample size (51,234 participants) and more comprehensive publications (including cross-sectional studies), providing sufficient data to detect the relationship. Third, we analyzed the association between PTH and hypertension and T2D from different aspects, involving the highest versus lowest PTH meta-analysis and linear dose-response meta-analysis.

The limitations in our study should be considered. First, the study involved cross-sectional data, which was weak in its ability to verify causality. We conducted a subgroup analysis based on different study characteristics, but no marked difference was observed in the overall effect size in the subgroups. Second, we found a moderate heterogeneity in this meta-analysis. The heterogeneity might derive from variations in sample size, study design, detect method and diagnostic criteria. Despite the heterogeneity, the summary ORs were consistent in the sensitivity analysis and most subgroup analyses. Third, there was publication bias in the meta-analysis to assess the risk of PTH and hypertension, however, the results did not change substantially by the trim-and-fill methods.

## Conclusions

In general, this dose-response meta-analysis shows a positive correlation between PTH levels and the risk of hypertension, but there is no statistical significance between PTH and T2D. The results of this study may provide some evidence for potential risk factors for hypertension and T2D, while proposing the thoughts that increasing dietary calcium intake may play a vital role in preventing hypertension.

### Electronic supplementary material

Below is the link to the electronic supplementary material.

Supplementary Material 1

Supplementary Material 2

## Data Availability

All relevant data used for the systematic review and meta-analysis are within the manuscript and its supporting information.

## Abbreviations

PTH: Parathyroid hormone
T2D: Type 2 diabetes
ORs: Odds ratios
CIs: Confidence intervals
