# Supplementary material for: Association of parathyroid hormone with risk of hypertension and type 2 diabetes: a dose-response meta-analysis
Source: BMC Cardiovasc Disord. 2024 Jan 3;24:13. doi: 10.1186/s12872-023-03682-1 (PMC10765621; doi:10.1186/s12872-023-03682-1)
Supplement: Supplementary file 2 — Supplementary Material 2 [file 12872_2023_3682_MOESM2_ESM.docx]

**Table 1S** Systematic literature review search terms and strategy

| Search terms for PubMed |
| --- |
| #1(“Parathyroid Hormone” [Mesh] OR “parathyrin” OR “PTH” OR “parathormone” OR “Parathyroid Hormone”) |
| #2 (“Hypertension” [Mesh] OR “High Blood Pressure” OR “High Blood Pressures” OR “Hypertension” OR “Blood Pressure” OR “Blood Pressures” OR “hypertensive”) |
| #3(“Diabetes Mellitus” [Mesh] OR “Glucose Metabolism Disorders” OR “Prediabetic State” OR “impaired fasting glucose” OR “Diabetes Mellitus” OR “type 2 diabetes” OR “type 2 diabetes mellitus” OR “prediabetes”) |
| #1 AND #2 AND #3 |
| Search terms for Web of Science |
| #1 TS= (“Parathyroid Hormone” OR parathyrin OR PTH OR parathormone OR “Parathyroid Hormone”) |
| #2 TS= (Hypertension OR “High Blood Pressure” OR “High Blood Pressures” OR Hypertension OR “Blood Pressure” OR “Blood Pressures” OR hypertensive) |
| #3 TS= (“Diabetes Mellitus” OR “Glucose Metabolism Disorders” OR “Prediabetic State” OR “impaired fasting glucose” OR “Diabetes Mellitus” OR “type 2 diabetes” OR “type 2 diabetes mellitus” OR “prediabetes”) |
| #1 AND #2 AND #3 |
| Search terms for Embase |
| #1 (“parathyrin” OR “PTH” OR “parathormone” OR “parathyroid hormone”) |
| #2 (“high blood pressure”/exp OR “high blood pressure” OR “high blood pressures” OR “hypertension”/exp OR “hypertension” OR “blood pressure”/exp OR “blood pressure” OR “blood pressures” OR “hypertensive”) |
| #3 (“glucose metabolism disorders” OR “prediabetic state” OR “impaired fasting glucose” OR “diabetes mellitus” OR “type 2 diabetes” OR “type 2 diabetes mellitus” OR “prediabetes”) |
| #1 AND #2 AND #3 |

**Table 2S** Characteristics of the individual studies included in this meta-analysis for parathyroid hormone and hypertension

| First author, publication year,  country | Study design, Follow up | Source of participant | Mean age | Gender | Cases/N | Dose(pg/ml) | ORs/RRs/HRs | Measurement of PTH | Definition of hypertension | Quality score |
| --- | --- | --- | --- | --- | --- | --- | --- | --- | --- | --- |
| Yao ,2016,  USA | cohort,  6years | The ARIC study | 56.1+5.6 | M/W | 1996/7504 | 24.9 | 1 | electrochemiluminescence immunoassay | Systolic BP ≥ 140 mmHg or diastolic BP ≥ 90 mmHg or the use of antihypertensive medications | 9 |
|  |  |  |  |  |  | 32 | 0.92 (0.80, 1.07) |  |  |  |
|  |  |  |  |  |  | 38.2 | 1.05 (0.91, 1.22) |  |  |  |
|  |  |  |  |  |  | 45.2 | 1.11 (0.96, 1.28) |  |  |  |
|  |  |  |  |  |  | 58.1 | 1.11 (0.96, 1.28) |  |  |  |
| Ballegooijen,2014,  USA | cohort,  9years | the MESA study | 45-84 | M/W | 1229/3002 | <33 | 1 | chemiluminescence assay | Systolic BP ≥ 140 mmHg or diastolic BP ≥ 90 mmHg or the use of antihypertensive medications | 9 |
|  |  |  |  |  |  | 33-44.2 | 1.00 (0.86, 1.17) |  |  |  |
|  |  |  |  |  |  | 44.2-65 | 1.09 (0.94, 1.28) |  |  |  |
|  |  |  |  |  |  | ≥65 | 1.27 (1.01, 1.59) |  |  |  |
| Taylor,2008,  USA | cohort,  10years | the Health Professionals | 40-75 | M | 142/481 | 26.3 | 1 | electrochemiluminescence immunoassay | self-reported hypertension | 7 |
|  |  |  |  |  |  | 33.6 | 1.22 (0.71–2.10) |  |  |  |
|  |  |  |  |  |  | 41.6 | 1.66 (0.99–2.77) |  |  |  |
|  |  |  |  |  |  | 56 | 1.83 (1.10–3.03) |  |  |  |
| Anderson ,2011，USA | cohort,  2years | the Healthcare system | 62.8±16.0 | M/W | 522/2617 | <15 | 1.50 (0.99-2.24) | chemiluminescence immunoassay | According to international disease classification | 6 |
|  |  |  |  |  |  | 15-75 | 1 |  |  |  |
|  |  |  |  |  |  | >75 | 1.95 (1.57-2.40) |  |  |  |
| Ballegooijen,2015, Netherlan | cohort,  6.4years | the PREVEND study | 44.5±10.8 | M/W | 1036/5066 | 4.24-26.69 | 1 | chemiluminescence assay | Systolic BP ≥ 140 mmHg or diastolic BP ≥ 90 mmHg or the use of antihypertensive medications | 9 |
|  |  |  |  |  |  | 26.79-32.82 | 0.98 (0.81-1.18) |  |  |  |
|  |  |  |  |  |  | 32.91-40.55 | 1.12 (0.93-1.35) |  |  |  |
|  |  |  |  |  |  | 40.64-197.09 | 1.11 (0.92-1.34) |  |  |  |
| Zhao,2010,  USA | Cross-sectional | the NHANES study | ≥20 | M/W | 2226/7228 | <27 | 1 | electrochemiluminescence immunoassay | Systolic BP ≥ 140 mmHg or diastolic BP ≥ 90 mmHg or the use of antihypertensive medications | 5 |
|  |  |  |  |  |  | 27-35 | 1.15 (1.00–1.32) |  |  |  |
|  |  |  |  |  |  | 35-43 | 1.19 (1.02–1.40) |  |  |  |
|  |  |  |  |  |  | 43-56 | 1.32 (1.11–1.57) |  |  |  |
|  |  |  |  |  |  | ≥56 | 1.32 (1.13–1.54) |  |  |  |
| Snijder ,2007,  USA | Cross-sectional | The LASA study | ≥65 | M/W | 958/1205 | 23.1 | 1 | immunoradiometric assay | systolic BP >140 mmHg and/or diastolic BP >90 mmHg and/or use of anti-hypertensive medication | 6 |
|  |  |  |  |  |  | 23.1-29.52 | 1.93 (1.30–2.86) |  |  |  |
|  |  |  |  |  |  | 29.61-40.08 | 2.00 (1.33–3.02) |  |  |  |
|  |  |  |  |  |  | 40.08 | 2.00 (1.31–3.06) |  |  |  |
| Kim ,2010,  Korean | Cross-sectional | the Chungju city area | 65+8 | M/W | 1026/1330 | 6.7-34.99 | 1 | chemiluminescence assay | Systolic BP ≥ 140 mmHg or diastolic BP ≥ 90 mmHg or the use of antihypertensive medications | 6 |
|  |  |  |  |  |  | 35.17-43.94 | 1.39 (0.88,2.20) |  |  |  |
|  |  |  |  |  |  | 43.94-53.66 | 1.01 (0.64,1.59) |  |  |  |
|  |  |  |  |  |  | 53.85-67.33 | 1.80 (1.08,3.02) |  |  |  |
|  |  |  |  |  |  | 67.8-279.13 | 1.23 (0.75,2.02) |  |  |  |

PTH: parathyroid hormone; M: men; W: women; OR: odds ratio; RR: relative risk; HR: hazard ratio; BP: blood pressure.; ARIC: Atherosclerosis Risk in Communities; MESA: Multi Ethnic Study of Atherosclerosis; PREVEND: Prevention of Renal and Vascular End-Stage Disease; NHANES : National Health and Nutrition Examination Survey; LASA: Longitudinal Aging Study Amsterdam.

| **Table 3S** Characteristics of the individual studies included in this meta-analysis for parathyroid hormone and type 2 diabetes | | | | | | | | | | |
| --- | --- | --- | --- | --- | --- | --- | --- | --- | --- | --- |
| First author, publication year,  country | Study design, Follow up | Source of participant | Mean age | Gender | Cases/N | DOSE pg/ml | ORs/RRs/HRs | Measurement of PTH | Definition of  type 2 diabetes | Quality score |
| Reis,2016,  USA | cohort,  6years | The ARIC study | 56.6 | M/W | 665/10100 | 25.2 | 1 | electrochemiluminescence immunoassay | nonfasting glucose level of ≥ 200 mg/dL; the use of glucose lowering medication and self-report | 9 |
|  |  |  |  |  |  | 32.6 | 0.98 (0.75, 1.28) |  |  |  |
|  |  |  |  |  |  | 39.1 | 1.00 (0.76, 1.30) |  |  |  |
|  |  |  |  |  |  | 46.5 | 1.07 (0.82, 1.39) |  |  |  |
|  |  |  |  |  |  | 60.8 | 1.26 (0.97, 1.64) |  |  |  |
| Anderson,2011,  USA | cohort,  2years | the Healthcare system | 62.8±16.0 | M/W | 338/5613 | <15 | 1.30(0.76-2.19) | chemiluminescence immunoassay | According to international disease classification | 6 |
|  |  |  |  |  |  | 15-75 | 1 |  |  |  |
|  |  |  |  |  |  | >75 | 1.40 (1.09-1.80) |  |  |  |
| Zhao ,2007,  USA | Cross-sectional | the NHANES study | ≥20 | M/W | 1002/3206 | <27 | 1 | electrochemiluminescence immunoassay | fasting glucose ≥100 mg/dl | 5 |
|  |  |  |  |  |  | 27-34 | 0.97(0.66–1.43) |  |  |  |
|  |  |  |  |  |  | 34-42 | 0.82(0.56–1.20) |  |  |  |
|  |  |  |  |  |  | 42-54 | 0.74(0.49–1.10) |  |  |  |
|  |  |  |  |  |  | ≥54 | 1.13(0.75–1.72) |  |  |  |
| Kim,2010,  Korean | Cross-sectional | the Chungju city area | 65+8 | M/W | 556/1330 | 6.7-34.99 | 1 | chemiluminescence assay | fasting glucose ≥5.6 mmol/l or if on drug treatment for elevated  glucose | 6 |
|  |  |  |  |  |  | 35.17-43.94 | 1.00 (0.68,1.48) |  |  |  |
|  |  |  |  |  |  | 43.94-53.66 | 0.92 (0.62,1.36) |  |  |  |
|  |  |  |  |  |  | 53.85-67.33 | 0.85 (0.57,1.27 |  |  |  |
|  |  |  |  |  |  | 67.8-279.13 | 0.85 (0.56,1.27) |  |  |  |
|  |  |  |  |  |  |  |  |  |  |  |
| Ballegooijen,2014,  USA | Cross-sectional | the MESA study | 45-84 | M/W | 182/3002 | <33 | 1 | chemiluminescence assay | a reported history of diabetes, use of any diabetes medication, or a blood glucose level ≥7 mmol/l | 6 |
|  |  |  |  |  |  | 33-34.2 | 1.01(0.69,1.47) |  |  |  |
|  |  |  |  |  |  | 44.2-65 | 1.14(0.78,1.67) |  |  |  |
|  |  |  |  |  |  | ≥65 | 0.90(0.49,1.67) |  |  |  |

*P*TH: parathyroid hormone; M: men; W: women; OR: odds ratio; RR: relative risk; HR: hazard ratio; BP: blood pressure; ARIC: Atherosclerosis Risk in Communities; NHANES: National Health and Nutrition Examination Survey; MESA: Multi Ethnic Study of Atherosclerosis.

**Table 4S** Quality assessment of included cohort studies

|  | | Newcastle-Ottawa Scale | | | | | | | | | | | |
| --- | --- | --- | --- | --- | --- | --- | --- | --- | --- | --- | --- | --- | --- |
| Author | |  |  |  |  |  |  |  |  |  |  |  |  |
| (Publication Year) | | selection | | | comparability | | | outcome | | | | total | |
|  |  | 1^a^ | 2^b^ | 3^c^ | 4^d^ | 5^e^ | 6^f^ | 7^g^ | 8^h^ | 9^i^ |  | |  |
|  | Yao et al. (2016) | 1 | 1 | 1 | 1 | 1 | 1 | 1 | 1 | 1 | 9 | |  |
|  | Ballegooijen et al. (2014) | 1 | 1 | 1 | 1 | 1 | 1 | 1 | 1 | 1 | 9 | |  |
|  | Taylor et al. (2008) | 1 | 1 | 0 | 1 | 1 | 1 | 1 | 1 | 0 | 7 | |  |
|  | Anderson et al. (2011) | 1 | 1 | 1 | 0 | 1 | 1 | 1 | 0 | 0 | 6 | |  |
|  | Ballegooijen et al. (2015) | 1 | 1 | 1 | 1 | 1 | 1 | 1 | 1 | 1 | 9 | |  |
|  | Reis et al. (2016) | 1 | 1 | 1 | 1 | 1 | 1 | 1 | 1 | 1 | 9 | |  |
|  | Anderson et al. (2011) | 1 | 1 | 1 | 0 | 1 | 1 | 1 | 0 | 0 | 6 | |  |

^a^ Representativeness of the exposed cohort; ^b^ Selection of the non-exposed cohort; ^c^ Ascertainment of exposure.

^d^ Demonstration that outcome of interest was not present at start of study.

^e^ Comparability of cohorts on the basis of the design or analysis (adjusted for age).

^f^ Comparability of cohorts on the basis of the design or analysis (adjusted for any other factor).

^g^ Assessment of outcome. ^h^ Was follow-up long enough for outcomes to occur.

^i^ Adequacy of follow-up of cohorts.

**Table 5S** Quality assessment of included cross-sectional studies

| Author | Cross-Sectional/Prevalence Study Quality | | | | | | | | | | | |
| --- | --- | --- | --- | --- | --- | --- | --- | --- | --- | --- | --- | --- |
|  |  |  |  |  |  |  |  |  |  |  |  |  |
| (Publication Year) | 1^a^ | 2^b^ | 3^c^ | 4^d^ | 5^e^ | 6^f^ | 7^g^ | 8^h^ | 9^i^ | 10^j^ | 11^k^ | total |
| Zhao et al. (2010) | 1 | 1 | 1 | 0 | 0 | 0 | 1 | 1 | 0 | 0 | 0 | 5 |
| Snijder et al. (2007) | 1 | 1 | 1 | 0 | 0 | 0 | 1 | 1 | 0 | 1 | 0 | 6 |
| Kim et al. (2010) | 1 | 1 | 1 | 0 | 0 | 0 | 1 | 1 | 0 | 1 | 0 | 6 |
| Zhao et al. (2007) | 1 | 1 | 1 | 0 | 0 | 0 | 1 | 0 | 0 | 1 | 0 | 5 |
| Kim et al. (2010) | 1 | 1 | 1 | 0 | 0 | 0 | 1 | 1 | 0 | 1 | 0 | 6 |
| Ballegooijen et al. (2014) | 1 | 1 | 1 | 0 | 0 | 0 | 1 | 1 | 0 | 1 | 0 | 6 |

*^a^* Source of Information; ^b^ Inclusion/Exclusion Criteria; ^c^ Time Period for Identity.

^d^ Subjects consecutive; ^e^ Evaluators Masked; ^f^ Quality Assurance Assessments.

^g^ Patient Exclusions; ^h^ Confounding assessed/controlled.

^i^ Missing Data; ^j^ Response Rates; ^k^ Follow-up.


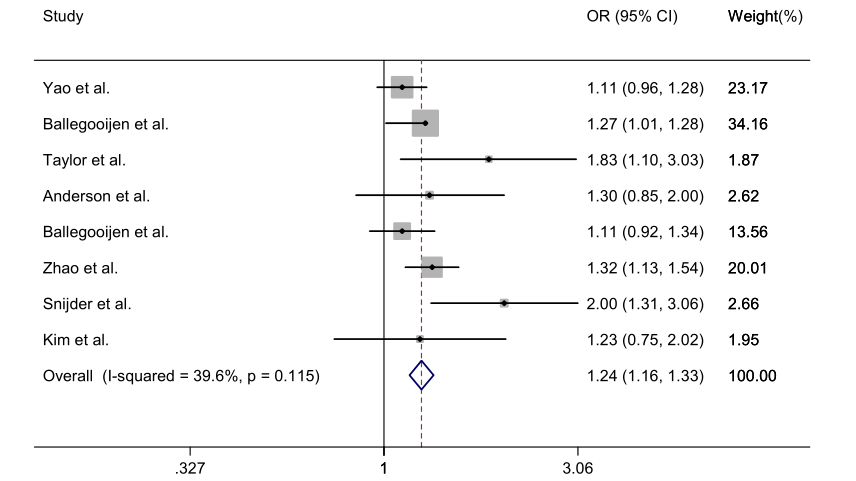


**Figure 1S** Risk of hypertension with parathyroid hormone (high/low)

**
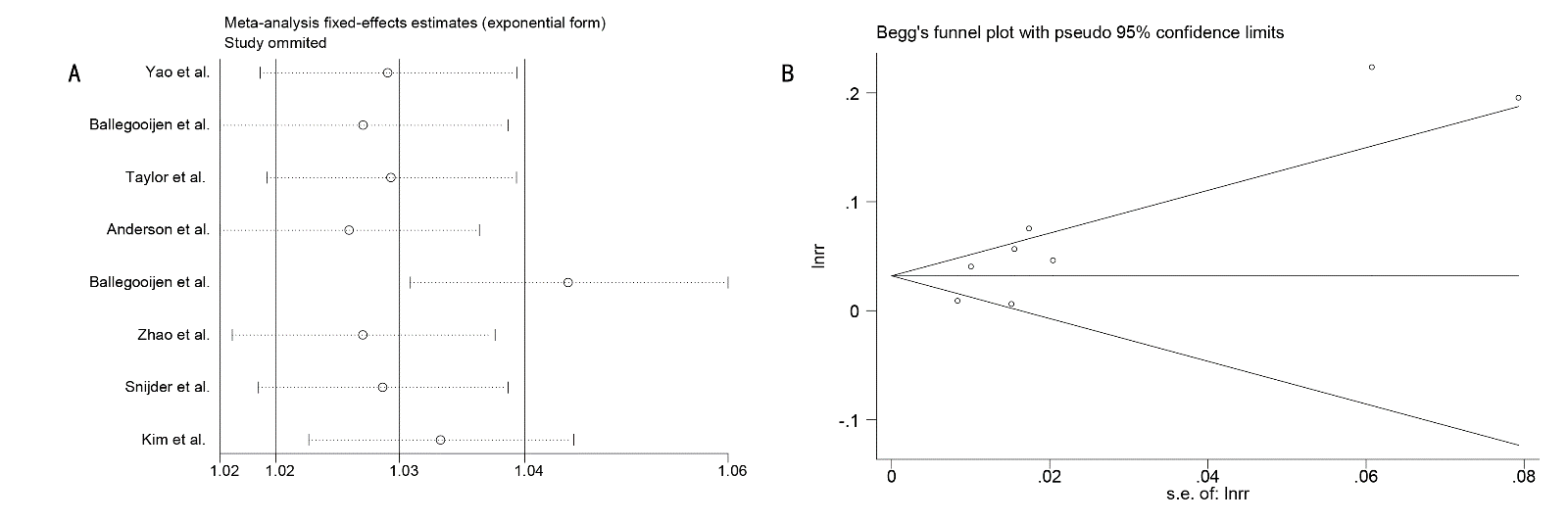
**

**Figure 2S** Sensitivity analysis (A) and Funnel plots (B) for meta-analyses assessing the association between parathyroid hormone and hypertension


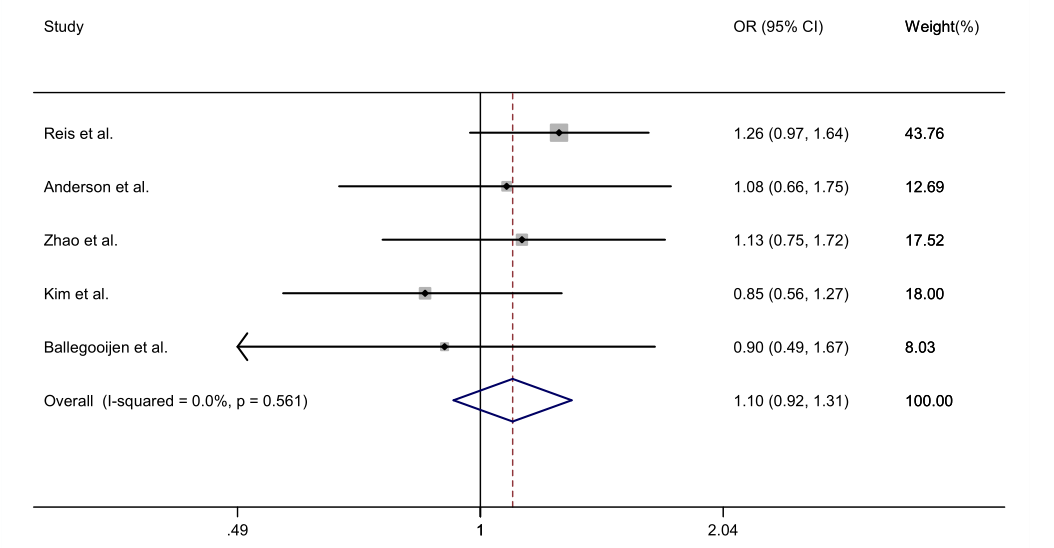


**Figure 3S** Risk of type 2 diabetes with parathyroid hormone (high/low)


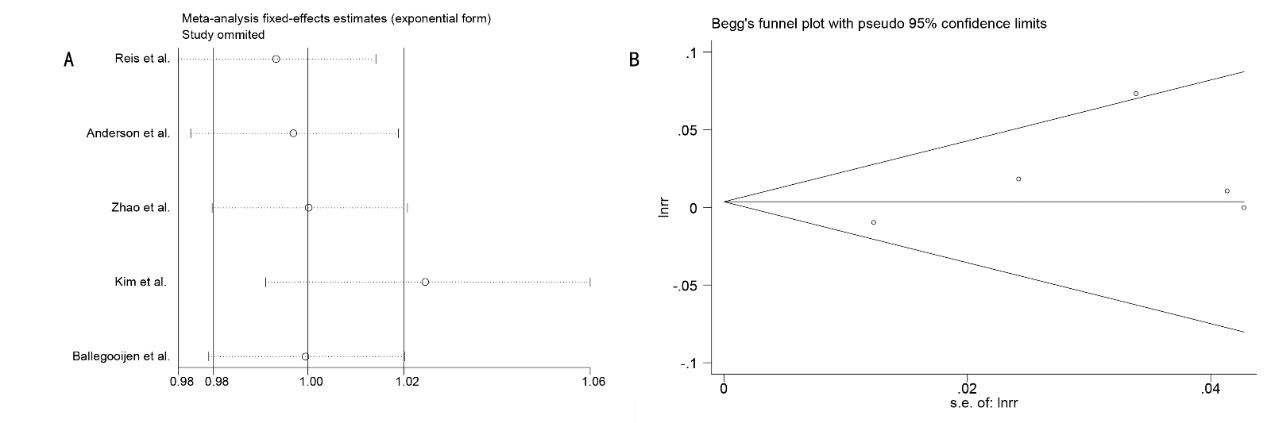


**Figure 4S** Sensitivity analysis (A) and Funnel plots (B) for meta-analyses assessing the association between parathyroid hormone and type 2 diabetes
